# Matrix Metalloproteinase 2 as a Pharmacological Target in Heart Failure

**DOI:** 10.3390/ph15080920

**Published:** 2022-07-25

**Authors:** Pricila Rodrigues Gonçalves, Lisandra Duarte Nascimento, Raquel Fernanda Gerlach, Keuri Eleutério Rodrigues, Alejandro Ferraz Prado

**Affiliations:** 1 Cardiovascular System Pharmacology and Toxicology Laboratory, Institute of Biological Sciences, Federal University of Pará, Belém 66075-110, PA, Brazil; pri.pharma.12@gmail.com (P.R.G.); duartelis18@gmail.com (L.D.N.); keuri13@yahoo.com.br (K.E.R.); 2 Department of Basic and Oral Biology, Faculty of Dentistry of Ribeirao Preto, University of Sao Paulo (FORP/USP), Ribeirao Preto 14040-904, SP, Brazil; rfgerlach@forp.usp.br

**Keywords:** MMP-2 inhibitor, cardiac dysfunction, ischemia

## Abstract

Heart failure (HF) is an acute or chronic clinical syndrome that results in a decrease in cardiac output and an increase in intracardiac pressure at rest or upon exertion. The pathophysiology of HF is heterogeneous and results from an initial harmful event in the heart that promotes neurohormonal changes such as autonomic dysfunction and activation of the renin-angiotensin-aldosterone system, endothelial dysfunction, and inflammation. Cardiac remodeling occurs, which is associated with degradation and disorganized synthesis of extracellular matrix (ECM) components that are controlled by ECM metalloproteinases (MMPs). MMP-2 is part of this group of proteases, which are classified as gelatinases and are constituents of the heart. MMP-2 is considered a biomarker of patients with HF with reduced ejection fraction (HFrEF) or preserved ejection fraction (HFpEF). The role of MMP-2 in the development of cardiac injury and dysfunction has clearly been demonstrated in animal models of cardiac ischemia, transgenic models that overexpress MMP-2, and knockout models for this protease. New research to minimize cardiac structural and functional alterations using non-selective and selective inhibitors for MMP-2 demonstrates that this protease could be used as a possible pharmacological target in the treatment of HF.

## 1. Introduction

HF is highly prevalent, affecting approximately 26 million people worldwide every year, with high rates of hospitalization and death [1]. According to the annual report on cardiovascular disease of the American Heart Association (AHA), the lifetime risk of developing HF is high, ranging from 20 to 45% after the age of 45 years. Estimates indicate that six million American adults aged 20 years or older have HF [2,3]. In Europe, the prevalence of HF is around 17.2 cases per 1000 individuals. It is an important public health problem with an average number of hospitalizations of 2671 per million people [4]. Furthermore, it is one of the most expensive syndromes in the US and Europe, consuming around 1–2% of the overall healthcare budget [5]. Global spending on CI is around US $108 billion per year [6].

**Figure 1:**
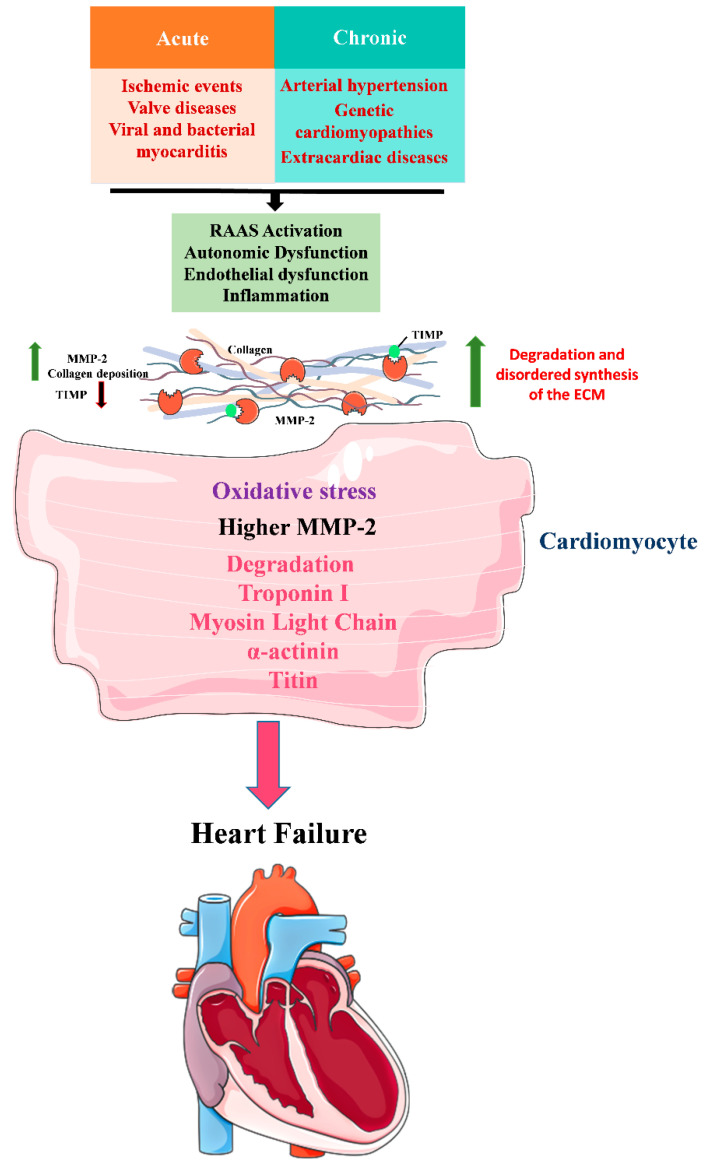
Cardiac remodeling in HF. HF occurs after an acute or chronic harmful event. The conditions that trigger this disease are hypertension, valvular diseases, genetic cardiomyopathies, myocarditis, extracardiac diseases and ischemia, generating autonomic dysfunction and activation of the renin-angiotensin-aldosterone system (RAAS), endothelial dysfunction and inflammation. In addition, the degradation and disordered synthesis of the extracellular matrix (ECM) occurs due to increased activity of MMP-2 and decreased activity of endogenous tissue inhibitors (TIMP), leading to collagen deposition and oxidative stress, causing degradation of components of the contractile apparatus, troponin I, light chain myosin, alpha-actinin and titin, promoting structural and functional changes; image elements from smart.server.com.

HF is an acute or chronic clinical syndrome that results in a decrease in cardiac output and an increase in intracardiac pressure at rest or upon exertion. In this condition, the heart is unable to pump enough blood to meet the metabolic needs of tissue [7]. HF can be determined according to the left ventricle (LV) ejection fraction (EF), characterized as the percentage of blood ejected from the LV with each systole. HF can be classified as reduced ejection fraction (HFrEF), HF with intermediate ejection fraction (HFiEF) or HF with preserved ejection fraction (HFpEF). Patients with HFrEF have a left ventricular ejection fraction <40%, with inadequate stroke volume and cardiac output as the primary manifestation. Patients with HFpEF have a left ventricular ejection fraction ≥50%, with impaired left ventricular relaxation. Patients with EF ranging from 41% to 49% were classified as HFiEF, presenting clinical characteristics similar to the population with HFpEF (Table 1). The New York Heart Association (NYHA) HF classification system, which stratifies the patient into classes I–IV, is based on the symptoms presented by the patient and the level of tolerated physical activity (Table 2) [8,9]. The etiology of HF stems from several conditions, and the leading causes are hypertension, valvular diseases, genetic cardiomyopathies, myocarditis, extracardiac diseases, and ischemia [10,11] (Figure 1).

The pathophysiology of HF is complex and results from an initial harmful event in the heart. The event can occur acutely (such as ischemic events, valvular diseases, and viral and bacterial myocarditis) or chronically (in arterial hypertension, genetic cardiomyopathies, and extracardiac diseases) and promotes functional and structural changes that compromise both systolic and diastolic blood pumping [7,9,12]. Diastolic dysfunction occurs due to structural changes resulting from fibrosis, promoting increased stiffness, decreased cardiac compliance, and hypertrophic cardiac remodeling, which causes an increase in LV filling pressure [12]. Systolic and diastolic electrical and mechanical asynchronies are related to the extent of diastolic dysfunction and exercise tolerance. Neurohormonal changes such as autonomic dysfunction and activation of the renin-angiotensin-aldosterone system are also implicated, as are endothelial dysfunction and inflammation. This makes HF heterogeneous and creates difficulties in choosing the therapeutic approach [13] (Figure 1).

In HF, cardiac remodeling occurs with degradation and disorganized synthesis of extracellular matrix (ECM) components. The ECM content is divided into fibrillar components (collagen, elastin, and reticular) and non-fibrillar components (glycoproteins and proteoglycans), which are responsible for tissue resistance and elasticity. Furthermore, their breakdown promotes functional changes. ECM metalloproteinases (MMPs) are proteases specialized in controlling the content of ECM [14,15,16] (Figure 1).

MMP-2 is a gelatinase constitutive of the heart that is considered a biomarker of patients with HFrEF and HFpEF. MMP-2 can digest components of the contractile apparatus, such as troponin I and light chain myosin 1, which contributes to the reduction in cardiac contractility [17]. At the transcriptional level, MMP-2 expression is controlled by transcription factors [18]. At the post-transcriptional level, MMP-2 activity and expression are regulated by inflammatory stimuli, oxidative stress, and alteration of the renin-angiotensin-aldosterone (RAAS) axis [19,20,21,22]. There is also a class of endogenous tissue inhibitors of metalloproteinase (TIMPs) that participate in the control of MMPs, and the balance between MMPs and TIMPs plays an essential role in the pathophysiology of heart disease [23,24] (Figure 1).

MMP-2 can be produced and secreted in the heart by cardiomyocytes, fibroblasts, endothelial cells and inflammatory cells present during the progression of HF. Although present in greater volume, cardiomyocytes are in smaller numbers than non-myocyte cells, which comprise 70% of the cells in cardiac tissue, most of which include fibroblasts. Cardiac fibroblasts maintain cardiac structural integrity by controlling cardiac extracellular matrix content. In addition, fibroblasts surround cardiomyocytes, and the myocyte function depends on the fibroblast. In pathological processes, cytokines and growth factors alter the fibroblast phenotype by increasing the secretion of ECM proteins that lead to fibrosis [25,26,27]. Fibroblasts and myofibroblasts are the central MMP-secreting cells in the heart and are indicated as a therapeutic target for the treatment of myocardial infarction, hypertension and HF [26].

Before introducing neurohormonal therapies in treating patients with HF, more than a third of deaths were attributed to sudden cardiac death. However, evidence-based clinical trials using neurohormonal treatments in patients not using a defibrillator showed a reduction in the rate of premature death. Furthermore, in addition to current pharmacological therapies, resynchronization devices, cardioverter-defibrillators and ventricular assist devices drastically reduced the risk of death. Indeed, HFrEF shows a different trajectory in response to drug therapy and cardiac resynchronization devices compared to HFpEF. However, both patient groups have benefited from available treatments, showing improvements in myocyte function, normalization of action potential duration, and improvement in mitochondrial energy metabolism. However, an unacceptable number of patients suffer impairment of functional capacity, low quality of life and early death due to HF. Thus, therapies that can stop or minimize the progression of HF continue to be challenging [8,9,28].

In this mini-review, we will highlight the participation of MMP-2 in cardiac alterations related to HF. Next, we will present some MMP inhibitors used in pre-clinical and clinical trials as a pharmacological tool for treating various diseases. Then we will address the use of MMP-2 inhibitors as an alternative treatment for HF in animal and human models. Finally, we will briefly explain the possible use of MMP-2 inhibitors and new technologies as an adjuvant treatment associated with standard therapy and their impact on the progression of HF.

## 2. Matrix Metalloproteinase 2 (MMP-2)

**Figure 2:**
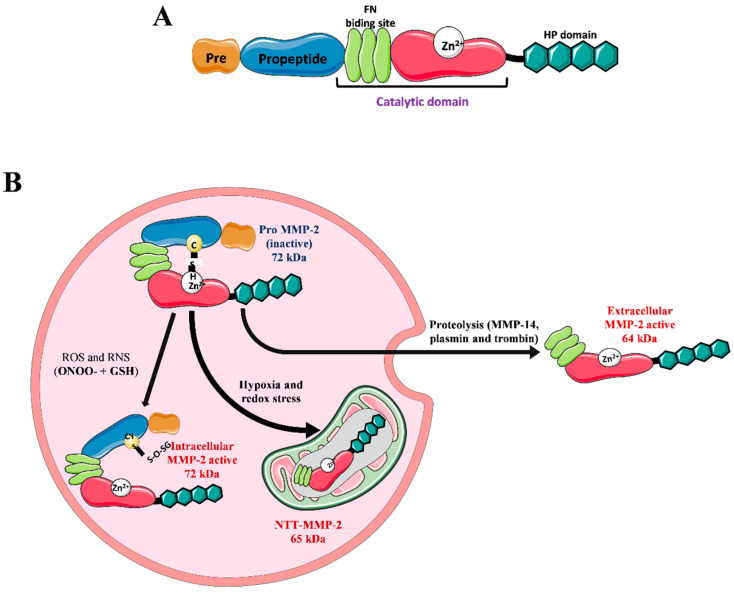
Structure, activation and isoforms of MMP-2. (**A**) MMP-2 has in its structure a signal peptide (Pre), a propeptide (Pro), a catalytic domain (having a zinc ion and three fibronectin repeats that confer affinity to collagen) and hemopexin (HP). (**B**) The inactive isoform of MMP-2 has a molecular size of 72 kDa (pro-MMP-2). Inactivity is guaranteed by a cysteine residue in the propeptide domain, which binds to Zn^2+^ in the catalytic domain, preventing the binding and proteolysis of substrates. The active intracellular isoform of MMP-2 has a molecular size of 72 kDa and occurs when ONOO^−^ or GSH reacts with ONOO^−^. The reaction product binds to the cysteine residue of the propeptide, which prevents it from complexing with the Zn^2+^ atom in the catalytic domain, allowing the catalytic domain to interact with substrates. The 65 kDa NTT-MMP-2 is constitutively active, formed under conditions of hypoxia and oxidative stress, and leads to activation of alternative MMP-2 promoters that do not translate the first 77 amino acids. This isoform is not secreted into the extracellular environment, found in mitochondria and cytosol. The extracellular isoform of MMP-2 with a molecular size of 72 kDa, activated by the proteolytic removal of the propeptide domain by MMP-14, thrombin and plasmin, produces an active isoform of 64 kDa. FN: fibronectin GSH: reduced glutathione; ONOO^−^: peroxynitrite; ROS: reactive oxygen species; RNS: reactive nitrogen species; NTT-MMP-2: truncated N-terminal isoform of MMP-2; image elements from smart.server.com.

MMPs are a family of proteases specialized in degrading ECM components, which have a highly homologous protein structure. Most have four basic domains: signal peptide, pro-peptide, catalytic, and hemopexin-like domains [29]. Based on their substrate affinity and structural organization, MMPs are commonly classified as collagenases, gelatinases, stromelysins, matrilysins, membrane MMPs, and others [30]. MMP-2 belongs to a group of gelatinases that have a unique catalytic domain among MMPs composed of a triple repeat of type II fibronectin, which forms a collagen affinity domain. This allows the binding and degradation of type IV collagen and denatured collagen (gelatin) [29] (Figure 2).

MMP-2 is encoded by a 27-kb gene that has 13 exons and 12 introns located on chromosome 16. The gene has consensus sequences for the transcription factors AP-2 and SpI [31]. The gene is transcribed into a 3.1-kb mRNA [32], which is translated into a 660-residue protein that contains a 29-residue signal peptide. This peptide is responsible for translocating MMP-2 to the endoplasmic reticulum, followed by secretion into the extracellular medium, giving rise to a latent enzyme of about 72 kDa [29,33] (Figure 2).

The absence of catalytic activity of the enzyme is maintained by the interaction between a sulfhydryl bond between a cysteine residue present in the pro-peptide and zinc at the catalytic site [34]. In the enzymatic activation, the catalytic site is exposed, which can occur through proteolysis of the propeptide by other proteases (MMP-2, plasmin, and thrombin) or through the interruption of the sulfhydryl bond by reactive species [35,36]. In proteolytic activation, an MMP-2 with a molecular size of 64 kDa is formed [29]. In the process of activation by reactive species, the molecular size of 72 Kda is maintained due to the permanence of the pro-peptide [36]. MMP-2 is expressed in most body tissues and modulates several physiological processes, such as cell migration, angiogenesis, and wound healing [29] (Figure 2). However, increased expression and activity of MMP-2 is involved in cardiovascular diseases such as atherosclerosis, aneurysm, hypertension, and HF [37,38].

## 3. Role of MMP-2 in HF

The knowledge of specific biomarkers used in the clinic to determine pathological states, define diagnoses or even prognoses are of great value since the symptoms of HF are often not pathognomonic, making diagnosis difficult. The main biomarkers to diagnose HF are the natriuretic peptides BNP and NT-pro-BNP [9,39]. However, BNP can be altered due to several factors such as kidney disorders, advanced age, obesity, diabetes, sepsis, Cushing’s syndrome and hyperthyroidism. Thus, the search for biomarkers that can help in the diagnosis and prognosis of HF becomes imperative.

MMP-2 can be considered a biomarker of HF, as higher plasma MMP-2 levels were found in patients with congestive HF, resulting from different etiologies (acute myocardial infarction, dilated cardiomyopathy and valvular disease). Higher levels of MMP-2 are correlated with patients with a worse prognosis for HF (NYHA class II–IV) [40], as well as an increased risk of death or hospitalizations for HF [41]. MMP-2 and TIMP-1 are higher in the plasma of patients with acute HF [42]. MMP-2 is considered the best biomarker among the other proteases in the ECM, as its levels varied only slightly during a temporal evaluation [43].

MMP-2 has also been considered a biomarker of LV remodeling in patients with HF who have suffered an acute myocardial infarction, with extensive areas of injury and decreased ejection fraction [44]. A study that evaluated control patients (who did not have cardiovascular disease), patients with LV hypertrophy without HF, and patients with diastolic HF and LV hypertrophy, showed that the dosage of MMP-2 and procollagen III N-terminal propeptide (PIIINP) together, were shown to be biomarkers that predict HFpEF better than NT-proBNP dosing alone [45]. MMP-2, MMP-9 and TIMP-1 have also been recognized as highly valuable biomarkers for predicting the risk of death in patients with HF [46].

In addition to being a biomarker, the participation of MMP-2 in the development of HF was suggested due to its role in the degradation of components of the myocardial matrix and the regulation of the fibrotic process, contributing to progressive dilation of the cardiac chambers, reduction in heart compliance and driving problems. In this context, MMP-2 is a key protease in the maladaptive remodeling process of the heart [20,40,41]

The role of MMP-2 in developing cardiac injury and dysfunction was demonstrated in a transgenic model of MMP-2 overexpression. Interestingly, this model showed that an increase in MMP-2 levels in the heart is sufficient to induce ventricular dysfunction, with myocyte hypertrophy, contractile protein lysis and cardiac fibrosis, even without a pathological process [47]. Furthermore, when subjected to ischemia and reperfusion injury, those animals that overexpress MMP-2 have higher infarction areas, lipid peroxidation and cardiac dysfunction compared to normal animals [48].

For a better understanding of the involvement of MMP-2 in pathological processes that lead to HF, the hearts of rats that suffered ischemia and reperfusion were evaluated, and there was an increase in the production of reactive species, including peroxynitrite (ONOO^−^) associated with MMP activation, before being secreted into the extracellular environment [36,49]. As a result, MMP-2 promoted proteolysis of contractile machinery proteins, including titin [50], troponin I [51], myosin light chain [52,53] and alpha-actinin [54]. In this experimental model, the disruption of the cell cytoskeleton by MMP-2 is involved in the decrease in myocardial contractility, with oxidative stress being the main factor related to the increase in MMP-2 activity. Thus, decreasing the oxidative stress or inhibiting the catalytic activity of MMP-2 may be a therapeutic target.

The redox imbalance in cardiomyocytes during ischemia also leads to the activation of alternative MMP-2 promoters, producing an N-Terminal Truncated isoform called NTT-MMP-2, constitutively active and present in mitochondria, altering energy metabolism and mitochondrial function and activation of the innate immune response [33]. In addition, overexpression of NTT-MMP-2 in mouse hearts results in LV hypertrophy, intense inflammatory cell infiltration, cardiomyocyte apoptosis and cardiac dysfunction [55].

Studies with knockout mice for MMP-2 were performed to confirm the participation of MMP-2 in HF after acute myocardial infarction. The absence of MMP-2 did not change the infarct area. However, the animals showed less LV dilation and increased survival than wild animals [56]. MMP-2 deletion was also beneficial in mice subjected to increased cardiac preload, in which they showed decreased myocyte hypertrophy and improved fibrosis and cardiac dysfunction [57].

On the other hand, inhibition of MMP-2 at below baseline levels can become an issue [58]. This was demonstrated in a preclinical study using MMP-2^−/−^ mice with cardiac overexpression of TNF-α showing decreased survival, LV contractile dysfunction, and increased infiltration of inflammatory cells of the myocardium [59]. Another study with *MMP-2*^*−*/*−*^ mice infused with angiotensin II showed that MMP-2 deletion did not affect the severity of hypertension but caused cardiac hypertrophy to develop earlier and to a greater extent than in wild-type animals [60]. Furthermore, clinical studies have shown that patients with loss of MMP-2 function due to mutations in the *MMP-2* gene are predisposed to a complex multisystem syndrome involving abnormalities of cardiac development [61,62].

The manipulation of MMP-2 genes helped us to confirm its participation in the pathophysiology of HF. However, when we analyzed the studies mentioned above, we realized that an exacerbated increase in activity or expression and the complete deletion of this protease is harmful during HF’s evolution. In this way, the ideal would be to modulate the levels and the activity of MMP-2 to prevent functional dysfunction caused by the remodeling of the heart. Therefore, the use of molecules that can inhibit MMP-2 may work as an effective treatment for the progression of HF.

## 4. The Development of Inhibitors for MMPs and Their Use as a Pharmacological Tool in Disease

The understanding of the role of MMPs in the pathophysiology of cardiovascular diseases, neurodegenerative disorders such as Parkinson’s and Alzheimer’s and cancer raised the hypothesis of the importance of regulating these prostheses as a way to stop changes in physiological processes such as angiogenesis, tissue remodeling, healing, migration cell, activation of signaling molecules and immunity [63]. Table 3 summarizes the different MMP inhibitors and their characteristics.

MMPs have endogenous inhibitors, including α2-macroglobulin, a protease secreted by the liver, that binds to MMPs in plasma, preventing them from degrading their substrates. Tissue MMPs are inactivated by TIMPs, with four members that make up this family: TIMP-1 to 4. TIMPs can inhibit all MMPs, but with different specificities for each one of them [64]. TIMPs have been used as a therapeutic tool in several diseases to modulate MMPs. However, without favorable results, possibly because they share similar pathways without directly interfering with each other’s role [63].

The first class of synthetic molecules used as inhibitors of MMPs was based on hydroxamate. These compounds were designed to mimic the natural peptide substrate (collagen) of MMPs associated with a group that can chelate the Zn^2+^ ion of the catalytic site, thus favoring broad-spectrum inhibition [65,66]. An example of this type of drug was batimastat, which could inhibit MMP-9 and slow the growth of solid tumors in preclinical trials [67]. In a double-blind prospective clinical trial, this drug was used to inhibit MMPs, as an adjuvant treatment in patients with small-cell lung cancer. However, it did not improve survival, decreasing the quality of life of patients who used it [68]. Another example of a hydroxamate-based drug was marimastat, a structural analog of batimastat, which showed favorable results in most preclinical studies in tumor models, thus serving as a basis for clinical trials [69]. A series of phase I, phase II and phase III tests were performed against solid metastatic tumors, with significant depletions in the increase in tumor markers being observed. However, in phase III studies, patients showed musculoskeletal toxicity, which may be associated with inhibition of ADAM and ADAMTS family members [66,70,71,72]. This class of hydroxamate-based inhibitors was able to inhibit several MMPs, including MMP-1, MMP-2, MMP-7 and MMP-9, showing benefits in preclinical trials but failed in clinical trials mainly due to their nonspecificity promoting combined inhibition of several MMPs resulting in musculoskeletal syndrome (arthralgia, myalgia and tendinitis) [63,66]. In addition, these drugs may have been introduced too late to modify a pathological condition, which could explain their failure in clinical trials. Therefore, first-generation hydroxamate-based molecules were discontinued after clinical trials failed.

However, a new generation of hydroxamate-based molecules was developed, presenting a sulfonamide and a zinc hydroxamate linking group and substituting an aryl group, generating a compound with more specificity to minimize the adverse effects of the previous generation triggered. Its development uses structure-activity relationship analysis (SAR), which helps identify molecular substructures related to the presence or absence of biological activity [63]. Cipemastat, used in treating patients with rheumatoid arthritis and osteoarthritis, is an example of this class. It was able to inhibit MMP-1, MMP-3 and MMP-9 more selectively. However, it did not prevent the progression of joint damage [66]. MMI-166 is selective for MMP-2, MMP-9 and MMP-14 and decreases the cellular invasion of cervical carcinoma in vitro. However, there was no suppression of the proliferation of tumor cells [66,73]. A recurring limitation in the use of these inhibitors was the premature metabolism suffered by the drug, leading to the loss of the hydroxamate group that binds with zinc. Despite the difficulties encountered in the therapeutic use of this new generation of inhibitors, there is still significant interest in developing drugs derived from hydroxamic acid, as these compounds are the most potent inhibitors of MMP available to date [63].

The search for molecules with lower metabolic lability and more stable bonds to the Zn^2+^ ion of the catalytic site led to the development of compounds derived from phosphoric acid, hydantoins and carboxylates, usually called non-hydroxamate MMP inhibitors. Next-generation MMP inhibitors have been designed with a variety of peptidomimetics and non-mimetics, not limited to mimicking their substrates [63]. One of the first non-hydroxamate MMP inhibitors developed was Rebimastat, a broad-spectrum inhibitor containing a thiol group that binds to zinc. Phase II clinical trials in early-stage breast cancer and a phase III study in lung carcinoma using this compound as adjuvant therapy discontinued treatment because patients experienced arthralgia consistent with MMP inhibitor-induced toxicity [74,75]. Another tanomastat inhibitor showed good tolerability and variable efficacy that depended on the timing of administration concerning disease progression [76,77]. Several biphenyl sulfonamide carboxylate-based MMP inhibitors have been designed to treat osteoarthritis by inhibiting MMP-13.

Substances such as polyphenols, flavonoids and carotenoids, obtained from natural products, can inhibit MMPs, with photoprotective and antioxidant properties. For example, *P. leucotomos* inhibits the expression of MMPs in epidermal keratinocytes and fibroblasts and stimulates TGF-β in skin fibroblasts by decreasing lipid peroxidation and oxidative stress [78,79]. Xanthohumol directly inhibits MMP-1, MMP-3 and MMP-9 while increasing the expression of collagen types I, III and V, fibrillin-1 and 2 in dermal fibroblasts [80]. Lutein prevents photoaging by inhibiting MMPs and oxidative stress, reducing epidermal hyperproliferation, expanding mutant keratinocytes, and mast cell infiltration in response to solar radiation [81,82]. The photoprotective activity presented by these compounds is probably associated with a decrease in the degradation of collagen fibers by MMP-1 and MMP-2 and of elastin fibers by MMP-2 and MMP-9 as well as by stimulating TGF-β in fibroblasts, which inhibits MMP-1 and stimulates collagen production [78]. Tetracycline antibiotics, such as doxycycline and minocycline, have an innate inhibitory capacity for MMPs [63,66].

To reduce off-target effects and to avoid broad inhibition of MMPs, due to their high structural homology, current inhibitors are being designed to target alternative, less conserved binding sites. Using crystallography and X-rays combined with computational methods allows the modeling of drug-protein interactions with inhibitors that bind to other sites. In addition, combining techniques with computational prediction revealed hidden sites in the structure of MMPs that can be explored for the rational design of new molecular effectors and therapeutic agents [63,66]

## 5. Use of Nonspecific and Specific Inhibitors for MMP-2 in HF

Preclinical studies demonstrate that a therapeutic approach to MMP-2 inhibition may be a promising strategy for treating patients with HF. ONO-4817 a selective inhibitor, has shown beneficial results in an ischemia and reperfusion model, improving contractile dysfunction, associated with decreased MMP-2 activity and titin proteolysis [50]. In addition, it showed promising results in attenuating LV remodeling and myocardial fibrosis in mice treated with doxorubicin, a drug used in cancer patients that is cardiotoxic and leads to HF, by increasing oxidative stress and MMP-2 activity [83]. Collectively these studies support the hypothesis that inhibiting MMPs by selectively using ONO-4817 has therapeutic potential, as this compound selectively inhibits MMP2, significantly decreasing the extent of lesions and disease severity. Furthermore, it cannot inhibit MMP-1, which has been associated with adverse effects triggered by hydroxamate-based inhibitors.

Antibiotics of the tetracycline class, such as doxycycline, which has an innate inhibitory capacity for MMPs, with greater specificity for MMP-2, MMP-9 and MMP-8, have also been used in models of cardiac injury. In preclinical trials, doxycycline prevented the conversion of concentric hypertrophy to eccentric hypertrophy of the LV during hypertension. This effect was associated with decreased MMP-2 activity and reduced troponin I and dystrophin proteolysis, thus improving the mechanical stability of cardiomyocytes and the contractile function [84]. On the other hand, doxycycline could not reduce scar thinning and compensatory LV hypertrophy, despite having decreased MMP-2 and MMP-9 activity in a model of acute myocardial infarction with LV dysfunction. These findings draw attention to the non-selective inhibition of MMPs in the initial healing phase after IM [85]. Doxycycline is an antibiotic capable of inhibiting MMPs at subantimicrobial doses and is currently the only FDA-approved MMP inhibitor for the treatment of periodontal disease [86]. In addition, it shows benefits in other conditions such as abdominal aortic aneurysm [87], arterial hypertension [88,89,90,91,92] and acute myocardial infarction [93].

Clinical studies that evaluated the effects of doxycycline on HF showed results dependent on the dose used and the etiology. For example, patients with acute myocardial infarction (40% of patients with HFrEF) were treated with doxycycline 100 mg as adjunctive therapy. They improved diastolic function and decreased the infarct area [94]. On the other hand, two randomized clinical trials that evaluated the effects of adjuvant treatment with doxycycline at a dose of 20 mg in patients with coronary artery disease and atherosclerosis showed no improvement in cardiac dysfunction parameters and sudden death outcomes [95,96]. The pathophysiological processes that trigger the damage and cardiac remodeling are closely correlated with the therapeutic response and the dose used since the preferential inhibition of specific MMPs are associated with the dose of doxycycline used as well time of therapy instituted.

The adverse effects of non-selective MMP inhibition are reinforced by the clinical study in patients with acute myocardial infarction and HFpEF, which evaluated the impact of the inhibitor PG-116800 (oral MMP inhibitor with an affinity for MMP-2, MMP-3, MMP-8, MMP-9, MMP-13 and MMP-14 and low affinity for MMP-1 and MMP-7). The PG-116800-treated group showed no improvement in heart function and death rates compared to the placebo. Unfortunately, this study was discontinued due to the development of musculoskeletal toxicity with no apparent benefit following administration of PG-116800 [97]. Therefore, we emphasize the importance of using inhibitors as an adjuvant therapy with greater specificity and fewer off-target effects.

In this way, using more selective inhibitors aimed at binding to alternative, less conserved sites can be a therapeutic strategy in HF, minimizing the adverse effects of nonspecific inhibitors. For example, TISAM, an N-sulfonylamino acid derivative, which selectively inhibits MMP-2, was used in a model of acute myocardial infarction, improving survival rate, preventing cardiac rupture and delaying post-infarction remodeling. These benefits were associated with decreased MMP-2 activity and macrophage infiltration into cardiac tissue [56].

Using chemical modeling to inhibit MMP-2 selectively, the inhibitors MMPI-1154, MMPI-1260 and MMPI-1248 were developed. MMPI-1154 and MMPI-1260 showed efficacy in reducing infarct size associated with decreased MMP-2 activity [98,99]. However, despite the potential of the TISAM, MMPI-1154 and MMPI-1260, these compounds have not been tested in other preclinical, experimental models of HF and clinical studies have not been conducted.

The selective inhibition of MMP-2 was also evaluated with siRNA technology. MMP-2 deletion in cardiomyocytes isolated from adult rats undergoing ischemia and reperfusion injury prevented contractile dysfunction associated with decreased MLC1/2 degradation [100]. The encapsulation of siRNA for MMP-2 in a hydrogel to improve cell penetration was also investigated in an in vivo acute infarction model. Positive effects on heart hemodynamics were observed, where the reduction in MMP-2 in cardiomyocytes led to the maintenance of cardiac output and ejection fraction [101]. Together, the studies that used compounds or technology of selective inhibition of MMP-2 reinforce that this protease can be a therapeutic target in the treatment of HF.

Clinical studies evaluating the effects of statins on MMP-2 inhibition (atorvastatin, rosuvastatin and pravastatin) in patients with HFrEF after acute myocardial infarction showed decreased serum MMP-2 levels associated with a reduction in the number of deaths and hospital readmission [102,103,104]. These studies did not assess whether these drugs directly inhibit MMP-2, but statins are known to decrease inflammation and oxidative stress [105,106], which may be related to the results found in reduced serum levels of MMP-2.

Drugs used in the treatment of hypertension, such as verapamil, carvedilol and trimetazidine, have shown positive effects on cardiac function and remodeling associated with decreased activity and expression of MMP-2 [107,108,109]. Low-dose carvedilol is cardioprotective and inhibits MMP-2 activity in an ischemia/reperfusion model [109]. Verapamil has been shown to reduce MMP-2 activity by decreasing oxidative stress and calpain-1 that regulates MMP-2 activity in a model of HF induced by hypertension [108]. At the same time, trimetazidine has reduced MMP-2 expression by decreasing oxidative stress in an animal model of myocardial infarction [107]. Table 4 summarizes the non-selective and selective MMP-2 inhibitors evaluated in preclinical and clinical studies of HF.

It is evident that MMP-2 plays an essential role in cardiac injury and HF development. Preclinical studies better explain the pathophysiological mechanisms involving extracellular matrix proteins. Clinical evidence indicates that selective inhibition of MMPs is optimal, as non-selective inhibition with MMP-inhibiting compounds is associated with loss of response and adverse reactions, possibly through inhibition of MMPs essential for body homeostasis. It is noteworthy that more clinical studies involving selective and non-selective MMP-2 compounds are needed, as the use of inhibitors for MMP-2 has shown promise as adjuvant therapy for HF in preclinical models. In addition, the use of drugs already used in the clinic can be an alternative for inhibiting MMP-2, having the advantage of safety in its use.

Selective inhibitors for MMP-2 tested in a multitude of diseases may be a plausible alternative in the treatment of HF, but the safety profile, possible adverse effects, and clinical results must be taken into account [110,111,112,113,114,115]. In addition, as a future perspective, the use of computational modeling as a tool can help predict the behavior of a molecule in non-living systems. Besides that, structure-activity relationship (SAR) analysis helps identify molecular substructures related to the presence or absence of biological activity. Finally, genomic engineering, such as clustered regularly interspaced short palindromic repeat (CRISPR), has a potential future in treating cardiovascular diseases, including HF.

## 6. Conclusions

Modulation of MMP-2 by non-selective or specific inhibitors has the potential to provide new directions for studying the mechanisms underlying various heart diseases, including HF. In addition, it has potential as a therapeutic tool for clinical practice and could have a significant impact on the development of new approaches to protect against cardiac remodeling and dysfunction in HF.

## Figures and Tables

**Table 1:** Definition of HF, according to left ventricular ejection fraction.

Classification	Left Ventricle Ejection Fraction (LVEF)	Main Cardiac Alterations (Ecodoppler)
HFrEF	<40%	Structural change and systolic dysfunction
HFpEF	≥50%	Structural change and diastolic dysfunction
HFiEF	41% to 49%	Structural change and diastolic dysfunction

HFrEF: heart failure with reduced ejection fraction; HFpEF: heart failure with preserved ejection fraction; HFiEF: heart failure with intermediate ejection fraction.

**Table 2:** New York Heart Association (NYHA) classification of heart failure based on symptoms and level of tolerated physical activity.

Class	General Description	Patient Symptoms
I	Asymptomatic	No limitation of physical activity; regular physical activity does not cause undue fatigue, palpitation and dyspnea.
II	Mild symptoms	Slight limitation of physical activity; comfortable at rest; activity results in fatigue, palpitation and dyspnea.
III	Moderate symptoms	Marked limitation of physical activity; comfortable at rest; regular exercise causes fatigue, palpitation and dyspnea.
IV	Severe symptoms	Unable to perform any physical activity without discomfort; HF symptoms at rest; if any physical activity is performed, the pain increases.

**Table 3:** Development of MMP inhibitors and their characteristics.

Class of MMP Inhibitors	Inhibitor (Alternative Names)	Characteristics
Endogenous inhibitors	α2-macroglobulin and TIMPs	It traps MMPs in the plasma, preventing them from degrading their substrates. It inhibits tissue MMPs and has four members: TIMP-1 to 4. TIMPs can inhibit all MMPs, but with different specificities.
Hydroxamate-based inhibitors	Batimastat and Marimastat	They are designed to mimic the natural peptide substrate (collagen) of MMPs. It targets the catalytic site of MMPs.
The new generation of hydroxamate-based inhibitors	Cipemastat and MMI-166	They were developed with a sulfonamide and a zinc-binding hydroxamate group, in addition to the substitution of an aryl group, generating a compound with more specificity. It targets the catalytic site of MMPs.
Non-hydroxamate inhibitors	Rebimastat and Tanomastat	They were designed with various peptidomimetics and non-mimetics, not limited to mimicking the substrate of MMPs. It targets the catalytic site of MMPs.
Inhibitors targeting alternative binding sites	BMS-275291 and specific MMP-13 inhibitor (provided by Pfizer, Ann Arbor, MI, USA)	Highly selective, unlike previous MMP inhibitors, because it does not bind to catalytic zinc ion and is not competitive for substrate binding. They target alternative, less conserved binding sites.

**Table 4:** Non-selective and selective MMP-2 inhibitors were evaluated in preclinical and clinical studies of HF.

Non-Selective Inhibitor	Species	Disease	Comments	References
Doxycycline	Rats	Renovascular hypertension with HF	Prevented the conversion of concentric hypertrophy to eccentric hypertrophy in the LV, associated with decreased MMP-2 activity and reduced troponin I and dystrophin proteolysis	[84]
Doxycycline	Mice	Model of acute myocardial infarction with HF	It has not reduced scar thinning and compensatory LV hypertrophy, despite having decreased MMP-2 and MMP-9 activity	[85]
Doxycycline (Adjuvant therapy)	Humans	Acute myocardial infarction (40% of patients with HFrEF)	Improved diastolic function and reduced infarct area	[94]
Doxycycline (Adjuvant therapy)	Humans	Coronary artery disease and atherosclerosis	There was no improvement in cardiac dysfunction parameters and sudden death outcomes	[95,96]
PG-116800 (Adjuvant therapy)	Humans	Acute myocardial infarction (HFpEF) with HF	No improvement in heart function and death rates Development of musculoskeletal toxicity	[97]
MMP-2 selective inhibitor				
ONO-4817	Mice	Ischemia and reperfusion model with HF	Shown to improve contractile dysfunction associated with decreased MMP-2 activity and titin proteolysis	[50]
ONO-4817	Mice	Model of doxorubicin-induced cardiotoxicity	Attenuated LV remodeling and myocardial fibrosis	[83]
TISAM	Mice	Model of acute myocardial infarction with HF	It improved survival rate by preventing cardiac rupture and delaying post-infarction remodeling	[56]
MMPI-1154, MMPI-1260 and MMPI-1248 (Chemical Modeling)	Mice	Model of acute myocardial infarction with HF	They showed inhibitory activity on MMP-2, associated with a reduction in the infarct area	[98,99]
siRNA for MMP-2	Mice	Ischemia and reperfusion model with HF	It prevented contractile dysfunction associated with decreased degradation of MLC1/2	[100]
Hydrogel encapsulated siRNA for MMP-2	Mice	Model of acute myocardial infarction	Improved cardiac output and ejection fraction	[101]
Statins (Atorvastatin, Rosuvastatin and Pravastatin)	Humans	Acute myocardial infarction (HFrEF)	Decreased serum MMP-2 levels are associated with a reduced number of deaths and hospital readmission	[102,103,104]
Antihypertensive drugs (Verapamil, Carvedilol and Trimethazine)	Mice and rats	Ischemia/reperfusion model; Model of HF induced by hypertension and Myocardial Infarction Model	Positive effects on cardiac function and remodeling associated with decreased activity and expression of MMP-2	[107,108,109]

## Data Availability

Not applicable.
